# Deciphering Autoimmune Pancreatitis, a Great Mimicker: Case Report and Review of the Literature

**DOI:** 10.1155/2015/924532

**Published:** 2015-01-29

**Authors:** Satya Allaparthi, Mohammed Sageer, Mark J. Sterling

**Affiliations:** Lahey Clinic, Burlington, MA 01805, USA

## Abstract

*Background*. Autoimmune pancreatitis (AIP) is an atypical chronic inflammatory pancreatic disease that appears to involve autoimmune mechanisms. In recent years, AIP has presented as a new clinical entity with its protean pancreaticobiliary and systemic presentations. Its unique pathology and overlap of clinical and radiological features and absence of serological markers foster the disease's unique position. We report a case of diffuse type 1 autoimmune pancreatitis with obstructive jaundice managed with biliary sphincterotomy, stent placement, and corticosteroids. A 50-year-old Caucasian woman presented to our hospital with epigastric pain, nausea, vomiting, and jaundice. Workup showed elevated liver function tests (LFT) suggestive of obstructive jaundice, MRCP done showed diffusely enlarged abnormal appearing pancreas with loss of normal lobulated contours, and IgG4 antibody level was 765 mg/dL. EUS revealed a diffusely hypoechoic and rounded pancreatic parenchyma with distal common bile duct (CBD) stricture and dilated proximal CBD and common hepatic duct (CHD). ERCP showed tight mid to distal CBD stricture that needed dilatation, sphincterotomy, and placement of stent that led to significant improvement in the symptoms and bilirubin level. Based on clinical, radiological, and immunological findings, a definitive diagnosis of AIP was made. Patient was started on prednisone 40 mg/day and she clinically responded in 4 weeks.

## 1. Introduction

In 1961, Sarles et al. [1] first described a case series of unusual pancreatitis associated with obstructive jaundice and hyper-g globulinemia. However, it was not until 1995 that Yoshida et al. [2] termed autoimmune pancreatitis to describe a case of diffusely enlarged pancreas with irregularly narrowing pancreatic duct that was serologically associated with hyper-g-globulinemia, anti-nuclear antibody positivity, and responsivity to steroid treatment.

Clinically, AIP is characterized by protean symptoms that have many features in common with pancreatic cancer. These symptoms include abdominal pain, obstructive jaundice, weight loss, steatorrhea, new-onset diabetes mellitus (DM), and elevated levels of serum tumor markers. Hardacre et al. in their single institutional study reported that about 2.5% of pancreatoduodenectomies were performed in AIP patients following a mistaken diagnosis of pancreatic cancer [3]. Since AIP responds extremely well to steroid therapy, it is of utmost importance that it be differentiated from pancreatic cancer to avoid unnecessary laparotomy or pancreatic resection. AIP is frequently associated with other systemic autoimmune diseases, such as rheumatoid arthritis, Sjogren's syndrome, sarcoidosis, and inflammatory bowel diseases.

The diagnosis of AIP is challenging as it closely mimics pancreatic cancer. We further report a case of a 50-year-old Caucasian woman who presented with epigastric pain, nausea, vomiting, and jaundice. Subsequent workup revealed AIP as the etiology of her symptoms, and she was treated effectively with steroids.

## 2. Case Presentation and Management

**Figure 1:**
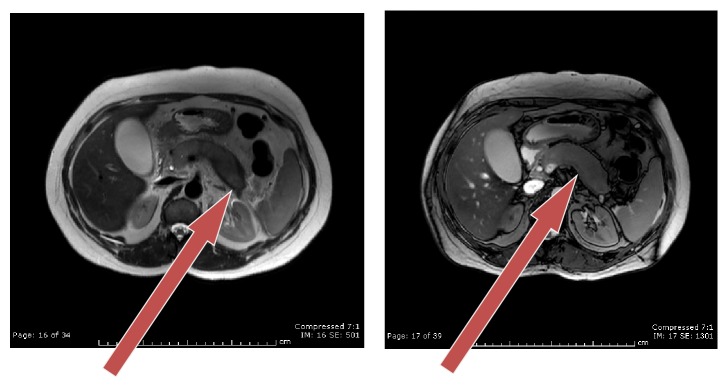
MRCP images arrow showing sausage-like pancreas in delayed phase.

**Figure 2:**
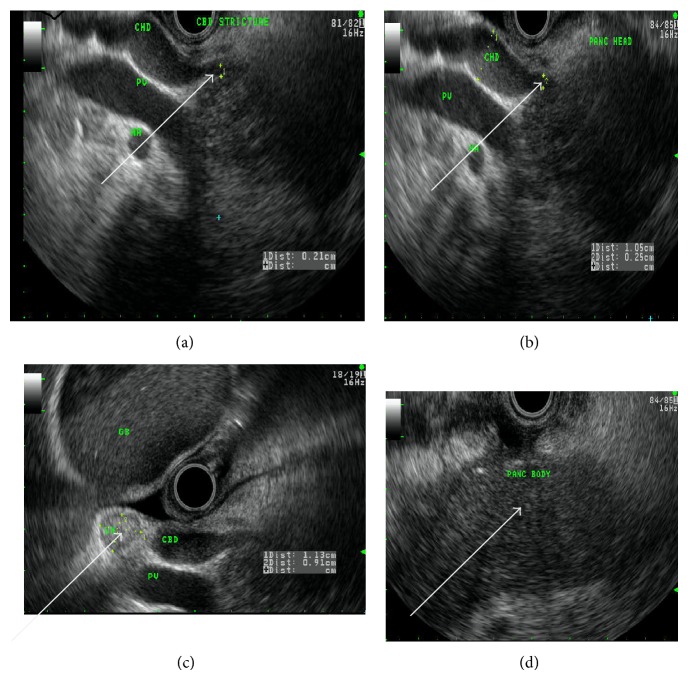
Endoscopic ultrasound images arrows showing (clockwise) (a) CBD stricture, (b) dilated CHD, (c) reactive lymph node, and (d) homogenous pancreatic body.

**Figure 3:**
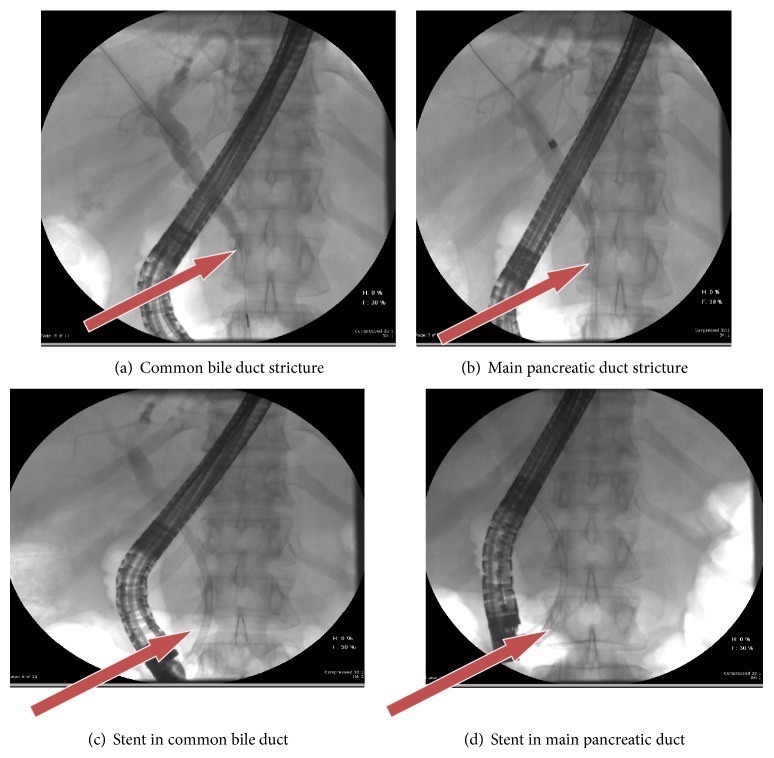
ERCP arrows showing CBD and MPD strictures pre stent insertion (a) and (b); post stent insertion (c) and (d) (clockwise).

A 50-year-old Caucasian woman presented to our hospital with 2-day duration of epigastric pain, nausea, vomiting, and jaundice. Her physical examination was unremarkable except for scleral icterus. An abdominal examination revealed epigastric tenderness without rebound. Laboratory investigations revealed hemoglobin 12.9 g/dL, white blood cell count 9.6/*μ*L, serum lipase 109 U/L, serum amylase 10 U/L, and total bilirubin 10.6 mg/dL (direct and indirect fractions 8 mg/dL and 2.6 mg/dL, resp.). Her liver enzymes were elevated (aspartate aminotransferase 110 U/L, alanine aminotransferase 131 U/L, and alkaline phosphatase 389 U/L). Tests for hepatitis A, B, and C are negative and found to have elevated immunoglobulin IgG Ab level of 765 mg/dl. Given these lab findings and clinical presentation, MRCP was further ordered which showed diffusely enlarged abnormal appearing pancreas with loss of normal lobulated contours. The pancreatic parenchyma is diffusely hypoenhancing, with focal hypoenhancement within pancreatic head, distal body, and tail (Figure 1). In view of her clinical presentation with worsening jaundice and MRCP findings, an endoscopic ultrasound (EUS) was done that revealed a diffusely hypoechoic and rounded pancreatic parenchyma with distal common bile duct (CBD) stricture and dilated proximal CBD and common hepatic duct (CHD) (Figure 2). Endoscopic retrograde cholangiopancreatography (ERCP) showed tight mid to distal CBD stricture that needed dilatation, sphincterotomy, and placement of stent that significantly improved her clinical symptoms and bilirubin level (Figure 3). She was started on tapering dose of prednisone 40 mg/day and 4 weeks after treatment she improved clinically and radiologically. Our ability to recognize AIP and differentiate it from pancreatic adenocarcinoma is aided by the use of international consensus criteria.

## 3. Discussion

Autoimmune pancreatitis is rare disease with a much lower rate of incidence than its principal differential diagnosis, pancreatic cancer. The overall incidence and prevalence are still unclear owing to lack of prospective natural history studies. Study series from Japan have reported the prevalence of autoimmune pancreatitis in a range between 5 and 6% of all patients with chronic pancreatitis of which 6–8% (0.82 per 100,000) had pancreatic resections performed for presumed pancreatic cancer [4, 5]. Moreover, in the last two decades, there has been an increase in the number of reports of autoimmune pancreatitis in the medical literature; however, the overall number of patients is still relatively small. Though this clinical entity was well described initially in Japan, a growing awareness of the condition has led to reports around the world. Hamano et al. [6] reported that serum IgG4 levels are specifically elevated in Japanese patients with AIP; however, in further reported case series by Hirano et al. [7] and Pezzilli and Corinaldesi [8], an increase of IgG4 levels in AIP cohorts has been also confirmed in Western and Eastern countries. Although the pathogenesis of the disease is unknown, current evidence strongly suggests that an autoimmune process has been implicated [9, 10]. Unlike most autoimmune conditions, AIP has a male predominance, with a male : female ratio of 2 : 1. The peak age of onset is the sixth and seventh decades [5, 9, 11].

The histopathological hallmark findings in patients with AIP include dense infiltration of T lymphocytes, IgG4-positive plasma cells, storiform fibrosis, and obliterative phlebitis in the pancreas; this form is termed lymphoplasmacytic sclerosing pancreatitis (LPSP) [12–14]. Of late, recent studies have provided evidence that there is another subtype of AIP with different histological findings named as idiopathic duct-centric pancreatitis (IDCP) that is more prevalent in Europe and the United States [15, 16]. Recent studies showed consensus that LPSP and IDCP are regarded as two distinct subtypes of AIP, and it has been proposed that LPSP be called “type 1 AIP” and IDCP “type 2 AIP” [17, 18].

In 2011, an international panel of experts met during the 14th Congress of the International Association of Pancreatology held in Fukuoka, Japan, and an international consensus diagnostic criterion for AIP was proposed [19]. According to these, AIP is classified into type 1 and 2. Five cardinal features of AIP are used: imaging of pancreatic parenchyma and ducts; serology; other organ involvement; pancreatic histology; and an optional criterion of response to steroid therapy. Each feature is categorized as a level 1 or 2 finding, depending on the diagnostic reliability (as shown in Table 1).

AIP should always be included in the differential diagnosis particularly in elderly presenting with obstructive jaundice and a pancreatic mass. Prior to initiation of therapy, it is of paramount importance to differentiate AIP from pancreatic cancer. Various strategies help differentiate the clinical, immunological, and radiological presentations in between AIP and pancreatic cancer. Obstructive jaundice induced by bile duct stenosis secondary to pancreatic cancer typically progresses steadily, whereas the jaundice of AIP in IgG4-related sclerosing disease sometimes fluctuates or, in rare cases, improves spontaneously [13, 20]. Elevated serum levels of IgG4 (>135 mg/dL) are seen in more than 90% of patients with AIP [6]. This is the most sensitive and specific diagnostic test for type I AIP, with 95% sensitivity, 97% specificity, and 97% accuracy for discrimination from pancreatic cancer [6]. Ghazale et al [21] in their study series of pancreatic cancer patients 13/135 (10%) noted that elevation of serum IgG4 levels alone cannot rule out pancreatic cancer, as only 1% of the above patients had elevated IgG4 levels >280 mg/dL, compared with 53% of AIP patients. Presence of other organ involvements such as bilateral salivary gland swelling, retroperitoneal fibrosis, and hilar or intrahepatic sclerosing cholangitis is highly suggestive of AIP rather than pancreatic cancer.

Radiological studies aid in differentiation based on the characteristic features on computed tomography (CT) and magnetic resonance imaging (MRI) that include diffuse or focal pancreatic enlargement, a peripancreatic capsule-like rim, enhancement at the late phase of contrast-enhanced images, and abnormal signal intensity on MRI. Diffuse enlargement of the pancreas and effacement of the lobular contour of the pancreas, the so-called “sausage-like” appearance, are a typical finding in AIP and are rarely seen in pancreatic cancer. On delayed phase of dynamic CT and MRI, enhancement of an enlarged pancreas is characteristic of AIP. As fibroinflammatory changes involve the peripancreatic adipose tissue, a capsule-like rim surrounding the pancreas is specifically detected in some AIP patients [22, 23]. The role of transabdominal ultrasonography in the diagnosis of autoimmune pancreatitis is not well established. Ultrasonographic images of the pancreas, obtained transabdominally, are rarely diagnostic of autoimmune pancreatitis. Furthermore, findings on ultrasonography may be similar for autoimmune pancreatitis and for other forms of acute and chronic pancreatitis.

The hallmark finding on endoscopic retrograde cholangiopancreatography (ERCP) in patients with autoimmune pancreatitis is diffuse or segmental attenuation of the main pancreatic duct (MPD), in contrast to the segmental stenoses often encountered with pancreatic adenocarcinoma. The other common findings are narrowing of the intrapancreatic portion of the common bile duct, irregular narrowing of extrahepatic bile ducts, and, less frequently, enlarged intrahepatic bile ducts [24]. It is of paramount importance to reliably distinguish AIP from PSC as making a reliable diagnosis is critical due to the often dramatic response of AIP related biliary strictures to steroid therapy in contrast to equivocal response seen in primary sclerosing cholangitis (PSC) and cholangiocarcinoma. The presence of a long, monomorphic stenosis of the intrapancreatic bile duct is suggestive of AIP, while band-like strictures, beading, or prune-tree appearance is most often found in PSC. Magnetic resonance cholangiopancreatography (MRCP) has become popular as a noninvasive method and it is becoming preferable to diagnostic ERCP. However, the narrowest MPD seen on ERCP cannot be visualized by MRCP due to the inferior resolution of MRCP compared with ERCP, so distinguishing between narrowing of the MPD in AIP and stenosis of the MPD in pancreatic cancer is not possible [25].

Endoscopic ultrasonography (EUS) emerged as a particularly important pancreatic imaging tool due to its ability to provide high-resolution imaging along with short working distances for transluminal pancreatic interventions. Though nonspecific, most common finding on EUS is diffuse or focal pancreatic enlargement inhomogeneous echo pattern, stranding and calcification [26]. EUS guided fine-needle aspiration or core biopsy of the pancreas may aid in the cytologic or histologic diagnosis; however, this approach to tissue acquisition was generally proven inadequate in providing a definitive diagnosis of AIP owing to a small sample size and lack of preserved tissue architecture and has not been evaluated in larger trials [26, 27].

## 4. Conclusion

In summary, we further report another case of AIP that reemphasizes the importance and various strategies of distinguishing it from pancreatic adenocarcinoma in order to avoid unnecessary surgical intervention. The concept of a “great mimicker” may be invoked and a heightened vigilance of AIP in one's differential diagnosis must be emphasized. As it is sometimes difficult to obtain adequate biopsy material from the pancreas, AIP is currently diagnosed based on careful consideration of a combination of characteristic clinical, serological, morphological, and histopathological features. More widespread use of pancreatic biopsy will aid in the diagnosis of autoimmune pancreatitis and provide a secure basis for the treatment with corticosteroids. Combined with a lack of prospectively validated clinical criteria that reliably establish the diagnosis, it is expected that the endoscopist will continue to play a central role in the diagnosis and management of AIP in the future.

## Figures and Tables

**Table 1:** Level 1 and level 2 criteria for type 1 AIP.

Criterion	Level 1	Level 2
Parenchymal imaging	Typical: diffuse enlargement with delayed enhancement (sometimes associated with rim-like enhancement)	Indeterminate (including atypical): segmental/focal enlargement with delayed enhancement

Ductal imaging (ERP)	Long (>1/3 length of the main pancreatic duct) or multiple strictures without marked upstream dilatation	Segmental/focal narrowing without marked upstream dilatation (duct size, <5 mm)

Serology	IgG4, >2x_upper limit of normal value	IgG4, 1-2x_upper limit of normal value

	a or b	a or b
	(a) Histology of extrapancreatic organs	(a) Histology of extrapancreatic organs including endoscopic biopsy of bile duct
	Any three of the following:	Both of the following:
	(1) Marked lymphoplasmacytic infiltration with fibrosis and without granulocytic infiltration	(1) Marked lymphoplasmacytic infiltration with fibrosis without granulocytic infiltration
	(2) Storiform fibrosis granulocytic infiltration	(2) Abundant (>10 cells/HPF) IgG4-positive cells
Other organ involvement (OOI)	(3) Obliterative phlebitis	
	(4) Abundant (>10 cells/HPF) IgG4-positive cells	
	(b) Typical radiological evidence	(b) Physical or radiological evidence
	At least one of the following:	At least one of the following:
	(1) Segmental/multiple proximal (hilar/intrahepatic) or proximal and distal bile duct stricture	(1) Symmetrically enlarged salivary/lacrimal glands
	(2) Retroperitoneal fibrosis	(2) Radiological evidence of renal involvement described in association with AIP

	LPSP (core biopsy/resection)	LPSP (core biopsy)
	At least 3 of the following:	Any 2 of the following:
Histology of the pancreas	(1) Periductal lymphoplasmacytic infiltrate without granulocytic infiltration	(1) Periductal lymphoplasmacytic infiltrate without granulocytic infiltration
	(2) Obliterative phlebitis	(2) Obliterative phlebitis
	(3) Storiform fibrosis	(3) Storiform fibrosis
	(4) Abundant (>10 cells/HPF) IgG4-positive cells	(4) Abundant (>10 cells/HPF) IgG4-positive cells

Reproduced with permission from 2012 Kamisawa, Tabata, Hara, Kuruma, Chiba, Kanno, Masamune, and Shimosegawa.

## Abbreviations

AIP: Autoimmune pancreatitis
MPD: Main pancreatic duct
CBD: Common bile duct
CHD: Common hepatic duct
LPSP: Lymphoplasmacytic sclerosing pancreatitis
IDCP: Idiopathic duct-centric pancreatitis
CT: Computer tomography
MRI: Magnetic resonance imaging
MRCP: Magnetic resonance cholangiopancreatography
ERCP: Endoscopic retrograde cholangiopancreatography
EUS: Endoscopic ultrasound-guided fine needle aspiration.
